# The Language of Vision*

**DOI:** 10.1177/0301006621991491

**Published:** 2021-02-14

**Authors:** Patrick Cavanagh

**Affiliations:** York University, Canada;; Glendon College, Canada;; Dartmouth College, USA

**Keywords:** Perception, language, attention, shapes/objects, spatial cognition

## Abstract

The descriptions of surfaces, objects, and events computed by visual processes are not solely for consumption in the visual system but are meant to be passed on to other brain centers. Clearly, the description of the visual scene cannot be sent in its entirety, like a picture or movie, to other centers, as that would require that each of them have their own visual system to decode the description. Some very compressed, annotated, or labeled version must be constructed that can be passed on in a format that other centers—memory, language, planning—can understand. If this is a “visual language,” what is its grammar? In a first pass, we see, among other things, differences in processing of visual “nouns,” visual “verbs,” and visual “prepositions.” Then we look at recursion and errors of visual grammar. Finally, the possibility of a visual language also raises the question of the acquisition of its grammar from the visual environment and the chance that this acquisition process was borrowed and adapted for spoken language.

There is a tradition that *Perception* publishes the Perception Lectures from each European Conference on Vision and Perception. Oops, it has taken almost 20 years for me to write this one up. Luckily, nothing much has happened since then to make it outdated. In fact, the reverse happened. I had been preoutdated—scooped—by of course Richard Gregory. In 1970, he had given a short talk on the BBC Radio’s Third Programme about an idea of his that there was—yes—a grammar of vision. The talk was transcribed in the BBC magazine, *The Listener*. I had not read his short note when I gave this lecture, but after my talk, he came up, flapping his prominent eyebrows enthusiastically, and said that he too had thought about these ideas and wasn’t it all fascinating. He didn’t mention, and I never found, the original but as I was finishing this paper, I asked Priscilla Heard if she knew of it, and within a day, she had sent me scans of three yellowed pages from *The Listener*. It was, indeed, fascinating and exactly what I had been working on. Not all was lost though, my paper has built on his original idea and serves, I hope, as a tribute to Gregory and the many insights that he gave us, some of them taking a while to bounce back into view. Let me summarize in a brief paragraph what he said to his audience on BBC Radio.

He was first of all puzzled that language had developed so rapidly in humans and intrigued by Chomsky’s (1965) recent idea that there was a deep structure underlying thought and language. He felt that *even* if language had started to develop a million years ago, it would be too little time for such a radical change in brain structure and function. Therefore, he proposed that Chomsky’s deep structure did not originally serve spoken language at all but instead had evolved many millions of years earlier to serve another end—to structure the world in order to see it. And always one for a bit of drama, he ended saying, “In the beginning was the grammar of vision—in the end came the word” (p. 244).

## Introduction

The visual system takes up a very large part of the brain—some say that as much as 30% of prime cortical real estate is specialized for visual processing in humans (Orban et al., 2004). This tells us that vision is important for our survival; language, touch, and audition, for example, get a far smaller slice of the cortical pie. So, what happens to all that visual information? Where does it go to next, how does it get there, and how is it interpreted? Many have pointed out that descriptions of the visual world must be exported to other modules in the brain where they can do useful work (e.g., Jackendoff, 1996; Mandler, 1999). For us to speak about what we see, some descriptions must be sent off to language centers; for us to catch a ball, recall a face, or read a book, descriptions of the visual scene must be reaching motor centers, memory centers, and reading processes.

**Figure 1.**
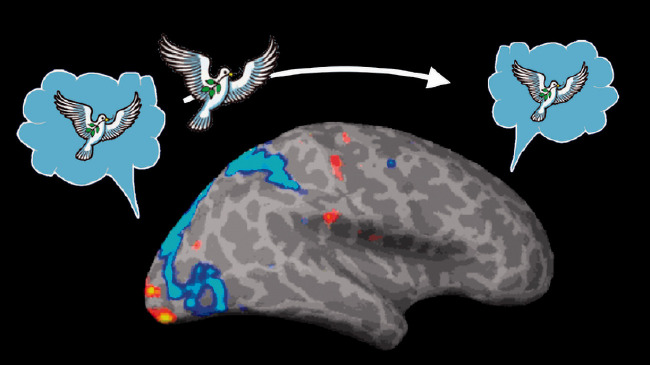
How does the visual system send messages to the rest of the brain? Sending pictures seems unlikely as that would require that the other brain modules have their own visual systems.

What are these descriptions that are sent from vision to the rest of the brain? We can imagine that for rapid motor control, say, maintaining our balance based on visual input, vision may be densely hardwired to the receiving area. These dedicated connections would support a high bandwidth, high-speed interaction that is specialized for the process being controlled. But what about visual input to more general-purpose cognitive processing (see Figure 1)? How does vision communicate with other modules in the brain?

**Figure 2.**
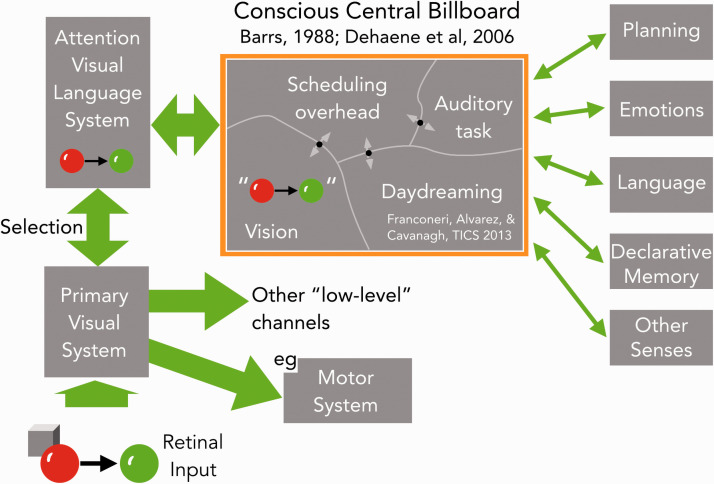
Visual language to central conscious “billboard.” Communications between modules could be on a one-to-one basis (Jackendoff, 1999) or, as here, mostly channeled through a common bulletin board or chat room (Baars, 1988; Dehaene et al., 2006) whose content is in awareness. Vision itself may have broadband, direct connections to some modules, for example, the motor system, to provide rapid visual guidance of actions. But the main path for visual descriptions to other modules here is via attention selecting and packaging events as a formatted description that can be understood by other modules. In the example here, the red and green balls that are about to collide are first selected from the input, ignoring the cube, then packaged as a description, shown here in quotes, that is posted on the billboard and available to the other modules of the brain.*Note.* Please refer to the online version of the article to view the figure in colour.

The messages that vision sends to other modules are not likely to be pictures or movies of what is happening. If they were, the receiving area would have to have its own visual system to interpret the pictures. Another alternative is that vision evolved a set of labels to denote objects, events, and scene layout. We will consider labels in more detail in a moment, but they do not get us very far. That leaves us with the possibility that vision packages the messages in a language that the other modules can decode. This would make a language of vision (as previously suggested by Gregory, 1970; Nicolas & MacNamara, 1995; Sereno, 1991) a type of “language of thought” or mentalese, a language or languages for communicating between the modules of the brain (e.g., Fodor, 1975; Jackendoff, 1996). Here, we will examine why a language would be a good choice, how it would be formatted, and what portion of the visual input would be packaged to send to other modules (Figure 2). And we will also ask what sort of evidence we can find for a language of vision, and finally, how a language of vision could be acquired by the brain.

The proposals in this article are conceptual in nature. Many will be difficult to test, and most are currently without any evidence in their favor other than their plausibility. Nevertheless, there is a need to address how vision communicates with the rest of the brain, and even if these proposals do not hold up, they are intended to trigger a search for alternatives that do, and ideas for testing them.

Many species communicate without using languages. Is there any reason to assume that vision needs a language? For example, nonhuman primates communicate using many different calls where one means a snake on the ground, another means eagle in the air, and so on, and some can even meaningfully combine these (Arnold & Zuberbühler, 2006). Could vision just be sending some complicated set of labels? Martin Nowak and coauthors examined the limits of communication by unordered labels and the advantages of grammars in several articles (Nowak et al., 2002; Nowak & Komarova, 2001). If each label can represent one thing, the number of possible labels will be restricted by memory—we can only learn so many labels. Let us say we can manage to learn Q labels that can be reliably transmitted under some noise considerations. However, if we allow two classes of labels, say, N and V, and we have some way to know the label’s type then we can combine labels in NV pairs. Now we can describe as many as (Q/2)^2^ things. If our memory limit were 2,000 labels, we can now describe 1,000,000 things. Quite an improvement. But it comes at a cost, the string of labels needs some format or grammar to mark which label is in one class and which in the other. If the grammar is not too burdensome, though, we still come out far ahead. The point made by Nowak and colleagues is that for communicating messages about a plausible number of things, language is not just an option; it is the only choice.

## So Really? A Language of Vision. How Does That Work? 

I have suggested that vision is composing messages to send to the other modules in the brain, and this is structured in the form of a language. O’Regan and Lévy-Schoen (1983) already proposed that the visual system’s representation of a scene was semantic in nature. That would be consistent with proposals that there is an internal language of thought for communicating between brain modules (e.g., Fodor, 1975). Psycholinguists like to point to this deeper coding level, or the deep structure of a language of thought (e.g., Chomsky, 1965; Lakoff, 1968), as a solution to the problem of ambiguous sentences where, for example, a word may have multiple meanings including be taken as either as a noun or a verb. Many famous examples have come from headlines: “Stud Tires Out”; “British Left Waffles on Falkland Islands”; “Prostitutes Appeal to Pope.” Certainly, a visual language would need to resolve the same issue, sending only the current interpretation of an ambiguous visual stimulus to the rest of the brain, rather than both. There may be several different modes of communication between modules with different formats and rules for each and so multiple languages of vision could be involved. However, the idea of one common format is appealing (Jackendoff, 1994).

Figure 2 lays out one such architecture for the traffic in formatted messages among modules, including vision. It relies on the idea of a central “blackboard” or “billboard” where messages from all modules are posted and read out. In the 1970s, this blackboard architecture was developed and implemented for large-scale projects in artificial intelligence (Erman & Lesser, 1975; Hayes-Roth, 1985; Nii, 1986). Bernie Barrs then proposed it as a central billboard for cognition (Baars, 1988), the chat room of the brain, and Stan Dehaene expanded on the concept as a Global Workspace (Dehaene et al., 2006). Both Barrs and Dehaene proposed that the content of the billboard is the content of consciousness.

This link to awareness is underlined in Figure 2 where the high-level messages that vision sends out to the rest of the brain are shown as the output of attentional selection. This is a plausible choice as attention and awareness have in common a severely limited capacity. Change blindness is perhaps the most compelling evidence for this. Changes in an image that are far above detection threshold can go unnoticed if attention is elsewhere (for review, see Simons & Rensink, 2005). Clearly, we attend to only a small subset of incoming visual information and the description that attention constructs for the selected material can then be the message that is shared with the rest of the brain. In other words, only one description is put together and shared with all the other modules, and this output is a rather low-bandwidth message. We can imagine that this message is what we experience as conscious vision if only because consciousness undoubtedly requires activity in many areas of the brain, so visual representations that become conscious are probably those shared outside strictly visual centers. If this proposal holds up, it offers some hope for decoding the language of vision as its content would be exactly the material that we can report as conscious visual percepts, as opposed to some obscure, hidden code. We need to determine only the grammar, the syntax, and the semantics of our conscious vision to decipher the internal language. We have our Rosetta stone right in front of us. It seems so simple.

## If Vision Has a Language, What Is it Made of?

**Figure 3.**
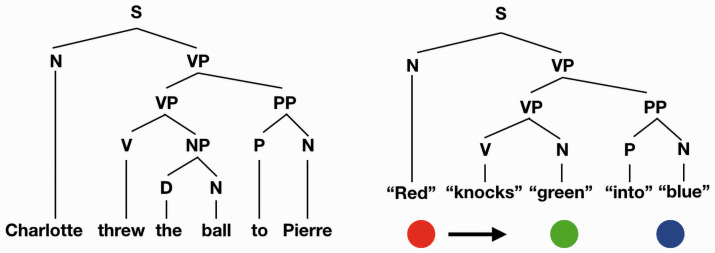
Left: A classic diagram of a sentence. Right: A similar diagram for a Michotte-like causal event that captures the structure of the event where the noun is an object and the verb is an action. The object, verb, and prepositions are in quotes to indicate that these are not verbal labels but labels in the visual language.

Spoken languages have verbs, nouns, prepositions, so why not visual language? Indeed, both visual and spoken languages have the job of describing the same world and so it is highly likely they share similar components. These superficial similarities show shared purpose, an example of convergent evolution. They do not imply that visual and spoken languages rely on any shared processes. In order for a visual language to construct descriptions of the world, we can imagine it needs elements such as objects (think, nouns), actions (verbs), and spatiotemporal relations (prepositions). It is a bit embarrassing to make such obvious analogies where we merely rename components of the visual world and claim, oh, look it can be a language. Nevertheless, let’s do so and see where we get. As the equivalent to the tree diagram of a sentence, we can then have a similar tree structure for an event where, for example, one ball rolls into another that then hits a third ball (Figure 3). Language is often jealously guarded as special skill that makes humans human. No other primate speaks. Starting with Hockett (1960), there has been a list of essential properties for communication to be considered a language, but this list is controversial (e.g., Wacewicz & Żywiczyński, 2015) and it is not fixed—Hauser, Chomsky & Fitch (2002) claim that only the last property, recursion is essential. Nevertheless, recent versions usually include the following four elements (e.g., Piper, 2012):

1. Compositionality/productivity: Combinations can produce an unbounded set of new and unique descriptions.
2. Arbitrariness: no link between symbol form and its meaning.
3. Displacement: Language is capable of describing things that are not present.
4. Recursion: embedding one component within a component of the same kind: that book that is on the chair that fell over.

As we go through a simplistic set of components for the language of vision, nouns, verbs, and so forth, we will be ticking off the boxes for these four hallmarks of what makes a language. Keep in mind that having these components may be essential for a communication system to qualify as a language, but they are not necessarily sufficient.

### Objects Are Nouns

**Figure 4.**
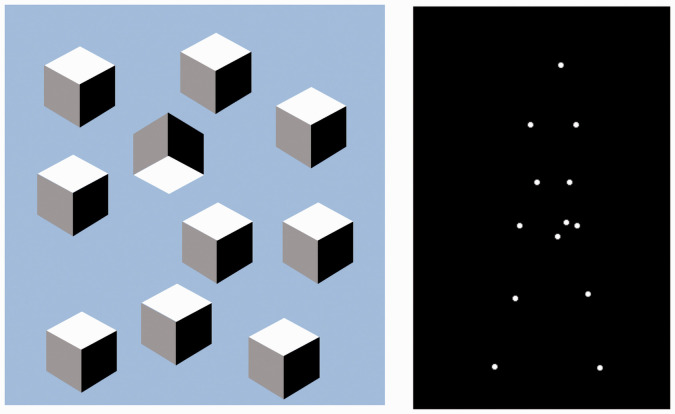
Left: Objects would be the nouns of vision, and some of them can be identified preattentively (from Enns & Rensink, 1990). Right: Familiar motion patterns would act as unitary verbs such as swim, flap, drop, hit, and so forth. An action sequence can take arbitrary subjects: Mary walks, François walks, the dots walk. These characteristic actions appear to require attention as visual search for a walker target is slow (Cavanagh et al., 2001).

Visual “nouns” maybe the output of ventral object recognition areas. The pattern of activity in the ventral area that designates the object is an arbitrary label though, not a little picture of the object. It does not look like what it refers to. It is just neurons firing. Activity patterns in early vision do have a 2D resemblance to the objects in the image on the retina (e.g., Tootell et al., 1988), but that pattern of activity is not the code for the object on the cortex any more than it is on the retina. In higher order areas that do code for objects, a single cell or group of cells may be active when their preferred target is present. One famous cell responded selectively to a variety of photographs of Jennifer Aniston (Quiroga et al., 2005) but obviously that cell does not resemble Jennifer Aniston. So vision has *arbitrariness*. I suggest that, in some cases, these object labels may emerge rapidly, based on the visual search literature where at least some objects can be identified in parallel (Figure 4, left panel) and the dual task literature where some objects may be detected in the near absence of attention (Li et al., 2002). However, most objects do not have this property and require attention to be identified slowly, one at a time (e.g., Hochstein & Ahissar, 2002).

### Actions Are Verbs

We might not immediately think of vision as having verbs because verbs typically have tenses. But vision could be considered to have tenses where, for example, any motion we see requires some comparison to the immediate past, and where the future is part of how we read intentions and expectations from visual cues. We most often associate this level of inference (intentions, expectations) to cognitive processes, so this proposal will require more analysis. Taking objects as nouns was pretty trivial; establishing that vision has verbs is a bit more involved.

I will suggest that familiar actions are the verbs of vision (Figure 4, right panel; Cavanagh et al., 2001). Rather than low-level motion signals such as leftward, rightward, up, and down, these would be high-level descriptions such as roll, bounce, soar, or break. Think of how a butterfly flutters or a frisbee sails, or a pencil drops and bounces, how an egg does not, or imagine the swing of a cottage door as it slaps closed. These are familiar actions that may act as single descriptors and, like regular verbs, they are reusable. It can be any frisbee sailing along or any pencil dropping. In the case of biological motion (Figure 4, right), a pattern as simple as a few dots appears to be walking. That means that the visual verb for walking is universal and can be applied to your friend walking, your mother walking, or a bunch of dots walking. Reusability is a key property of verbs: They take a subject (a noun or object) and that subject then is described as executing the verb’s action. This is the basis of *compositionality* that allows combinations of verbs and nouns to describe an unbounded set of events. The characteristic motions of familiar objects such as a butterfly in flight also contribute to the recognition of these objects. In return, once the object and its stereotypical motion are recognized, knowledge of that motion can support the continuing percept. Like the first notes of a familiar tune, our knowledge can help us follow the remainder of the melody, filling in missing notes. Selfridge (1959) had argued that shape recognition was supported by legions of “daemons,” each of which searched for its matching static pattern in the scene and signaled when it showed up. This idea is extended here to a dynamic version where myriads of characteristic actions, the verbs of vision, identify their matching motion patterns in the input, helping to recognize the object and maintain its continuity of action in noisy input, and then give the activity a compact label, like “walking” as part of the description sent out from vision.

### Tenses of Visual Verbs

**Figure 5.**
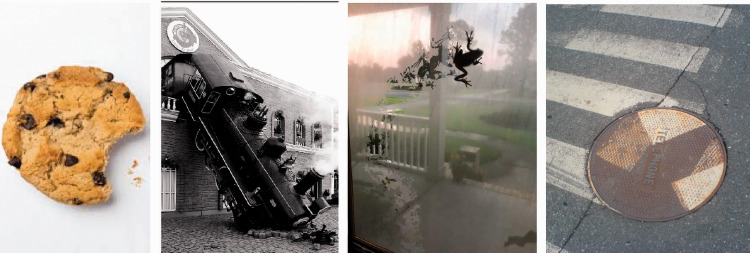
Past tense. Left: When we see this cookie, we also see its past. It seems immediately obvious. Similarly, a train has unfortunately left its normal track, and its past history is clearly seen. Right: In contrast, the past history requires more cognitive inference than visual in these examples. A frog leaves a trail on the misty window glass, and a manhole cover has been removed after painting and replaced at an angle.

**Figure 6.**
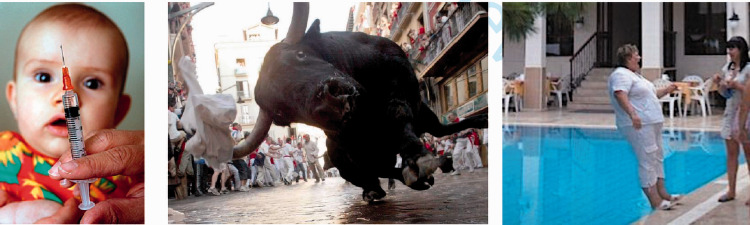
Future tense. Left: There will be some distress in a moment. Middle: This will not end well. Right: The woman will soon be wet.

It seems that vision is pretty immediate—we see what is happening right now, whereas past and future tenses would refer to events that are not currently visible. But what we see is often a construct, integrating past states and future outcomes (Figures 5 and 6). For example, many objects carry evidence of past history: half-eaten cookies, damaged cars, cigarette butts, or a bruised arm. These transformations of canonical shapes are a type of visual past tense (Figure 5, left), a record of the cause of the current shape (e.g., Choi & Scholl, 2006; Fleming & Schmidt, 2019; Leyton, 1989). Is this interpretation of a past history a visual or cognitive inference (see Box 1)? Here, Chen and Scholl (2016) showed that some shape distortions could trigger perceptions of motion. They argued that the motion response was specifically visual and, therefore, that vision can reconstruct causal history from static shapes without relying on any cognitive inference. Based on their finding, I will suggest that in some cases these visible deformations act as the past tense of visual verbs—the cookie was eaten (Figure 5, left). However, the past leaves many traces, and it is likely that only a few of them are directly decoded by the visual system and added to the object’s description to indicate past state of the object. For example, the frog’s path (Figure 5, middle right) is a record of a past event, but it takes a moment to realize what happened here. Based on the delay in understanding, intuition argues that this is more of a cognitive deduction.

Much of the information in a visual scene is simply in the present tense. But when we see something in motion, it does involve a comparison of the immediate past and the present. That makes it the visual equivalent of the present progressive tense—he is walking; they are eating; the bird is flying.

The future tense again raises the question of how much of the message is computed by the visual system and how much is a later cognitive deduction, a question that will come up again when we consider visual grammar. Figure 6 shows some examples of how a static image predicts future events. These convey a sense of foreboding and a gut feeling of wanting to step back (Figure 6, left), or jump away (Figure 6, middle), or reach out (Figure 6, right), that is felt with no cognitive effort. Again, our visual systems cannot decode the future consequences of all scenes. Perhaps only those situations that clearly predict immediate consequences get tagged with the likely future outcome. Part of predicting future events depends on understanding the intentions of others. Heider and Simmel’s (1944) demonstration of the assignment of intentions to animated dots has intrigued us for decades. It suggests that the visual system may assign goals to agents in the scene although it remains to be shown that these assignments emerge in the visual system and not later.

### Prepositions

**Figure 7.**
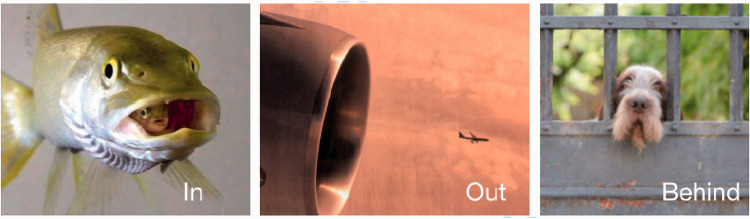
Prepositions. In vision, prepositions represent the spatial and temporal relations among objects and actions. The preposition “behind” has special importance because it is equivalent to the visual phenomenon of completion—we represent a whole dog behind the gate, not just a disembodied head. This ability to reference something that is not present is a key property of language.

Prepositions capture the spatial and temporal relations between elements: before, on, between, inside, outside, above, behind (Figure 7). One preposition plays a critical role in the claim of a language of vision. That is *behind*. The example in Figure 7 on the right demonstrates the point that when we see a dog behind a gate, we complete the missing parts of the dog (called amodal completion), and we would be surprised if the gate were opened and the dog had no body. The reason that this is relevant to the question of language is that languages must have the property of *displacement*—they can reference things that are not present in front of us. Clearly, the missing parts of the dog are generated in its description that is shared with the rest of the brain. Vision has *displacement*.

**Figure 8.**
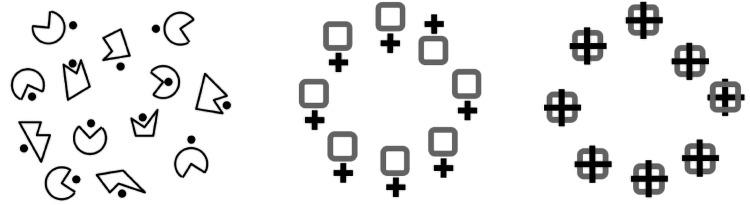
Visual search for spatial relations. It is difficult to find the one form with the dot inside instead of outside (Treisman & Gormican, 1988) or the square with the plus above not below (Logan, 1994, 1995), or the square with the cross behind rather than in front (Moore et al., 2001). Spatial relations are not determined preattentively but require scrutiny.

In vision, these spatial relations are probably not explicit until we pay attention to both elements being compared. This is the point of Steve Franconeri’s work on deriving the relation between objects by paying attention to each in turn (e.g., Franconeri et al., 2012). Certainly, the visual search literature backs this up as finding a target defined by a spatial relation is quite slow (Figure 8). The point is that it takes time and attention to build up a description not only of who is doing what to whom but also of the relations between elements in the scene. There are several studies on how we compare a linguistic description of a spatial relation to its visual counterpart (Clark & Chase, 1972; Jackendoff, 1983; Logan, 1994, 1995; Roth & Franconeri, 2012). In all these cases, it is effortful to verify the equivalence of the visual and linguistic description (e.g., star above square) in agreement with the visual search evidence that these relationships are not expressed preattentively.

### Causality

Causality (Michotte,1946/1963) is a determination of who did what to whom, and in that sense, it takes an action and assigns the subject and the object for the action. Many would expect that causality would be a high-level cognitive inference, but we now have evidence that some types of causality are worked out in the visual system. Some years after this talk was given, two studies demonstrated evidence that causality can be a visual computation (Kominsky & Scholl, 2020; Rolfs et al., 2013). These studies showed that adaptation to a series of causal events where one disc hit another (collision or launching) made subsequent ambiguous events appear less causal. Most important, this adaptation was retinotopic (Rolfs et al., 2013)—when the adaptation was to the left and right of fixation, the effects were seen there and not above and below (and vice versa). The area where the adaptation had an effect also moved when the eyes moved. These results strongly suggested that at least this form of causality was determined in the visual system and was not a cognitive inference. Any adaptation of cognitive inference would have had a general effect throughout the visual field. This evidence that vision itself processes and signals causality is an important step in the claim that vision understands who did what to whom, a critical precursor to sending that description out in a language of vision.

### Recursion

**Figure 9.**
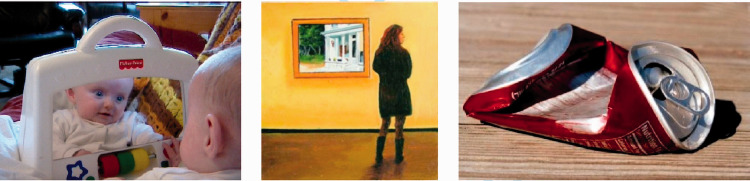
Recursion. When one description is embedded within another, it is a recursive structure. Mirrors and paintings within paintings provide two sets of descriptions of visual space, one embedded within the other. A deformed familiar shape has a history embedded in its description.

Recursion has had a privileged status in differentiating language from nonlanguage. It consists of embedding a constituent in a constituent of the same type. In spoken language, a relative clause inside a relative clause is recursive (the man who was climbing the steps that you sat on last night). This then allows us to do so endlessly (e.g., the man [who was climbing the steps [that you sat on the night [that we chose to go to the movie [that you liked so much]]]]). According to Hauser et al. (2002), recursion is the only thing that separates language from nonlanguage. This extreme point of view is controversial (see Pinker & Jackendoff, 2005), and, moreover, there is a claim that there is at least one spoken language, Pirahã, that does not use recursion (Everett, 2005, 2009). However, this debate ends up in the linguistics world, there is no lack of recursion in vision (Figure 9). We see embedding of visual scenes within visual scenes whenever there is a picture in a picture, in a photo, or a painting within a painting within a photo (Figure 9, middle), each scene with its own pictorial space. Another form of embedding is the history seen in a deformed object (Figure 9, right panel). Vision is deeply *recursive*. Being recursive on its own does not make vision a language, but it is, nevertheless, one piece of the larger argument.

## Visual Grammar

The real engine underlying language is its grammar—the way components are composed and combined that identifies the class and properties of each component. Machine vision, for example, often has explicit grammars for specifying the structure of images (e.g., Fu, 1974; Savova & Tenenbaum, 2008; for review, see Zhu & Mumford, 2006). These structured descriptions of objects have been applied to human vision as well (e.g., Biederman, 1987; Marr & Nishihara, 1978), but they stop at descriptions of static structures without attempting to handle actions within the scene.

Some authors have suggested that the descriptions passed from vision to cognition are in the form of propositions (Logan & Zbrodoff, 1999; Miller & Johnson-Laird, 1976), structured descriptions of the relations among a set of elements along with the truth values of those relations for the scene in question. Propositions are an awkward choice for capturing continuous events—graphs might be better—but even so, these suggestions just avoid the question of what a grammar might be by simply substituting one description for another. There are no rules here yet, and it may be hard to determine what rules would underlie a grammar for human vision. In fact, the rules and the nature of the formatted message may be so foreign to us that we cannot imagine, yet, what they may be. As a result, I will avoid proposing what the rules might be.

**Figure 10.**
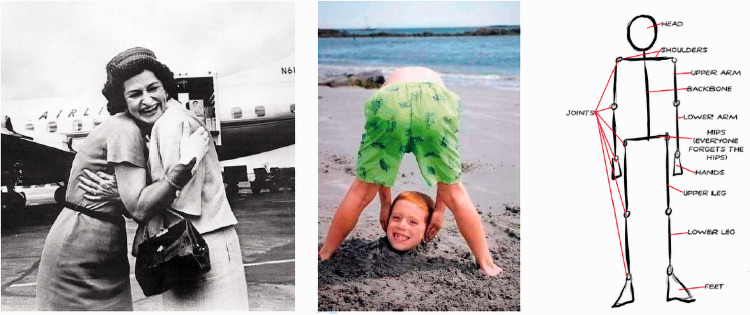
What would errors of visual grammar be? These two images on the left might seem puzzling as in each case, it is not clear who owns the head. The visual system appears to have a schema for body parts that it tries to complete (e.g., far right). It fails on the left because there are two bodies that could equally well own the head. On the right, it feels as if the head in the sand belongs to the body above it. This sense of searching for a connection may be evidence of a visual object syntax that is being broken in these two images.

**Figure 11.**
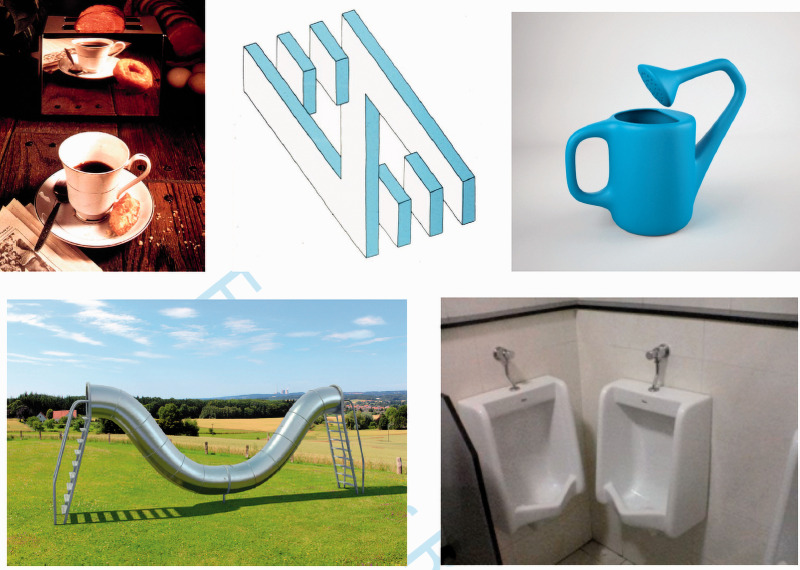
Errors of visual grammar or design glitches discovered cognitively? Clearly, there are many impossible scenes, or uncomfortable designs, that are noticed as impossible or uncomfortable by cognition, not vision. The reflection in the toaster does not match the scene in front of it; the 3-D structure in the center panel appears to be impossible; it is not possible to pour water from the container; children trying this slide will not be able to get out; the two plumbing fixtures break some social rules for appropriate use.

Nevertheless, even without knowing what the rules are, we can take a reverse approach and ask what ungrammatical vision would be. We often only know what the rules of spoken grammar are when we notice errors, and we can look at vision the same way. So, what would ungrammatical vision look like? Perhaps an impossible event or magic (Gregory, 1970, made a similar suggestion). Whatever the case, we can imagine that an image that challenges the rules of visual grammar, if there is one, would give us a pause when we first see it. We should therefore look for pictures where something seems at first implausible or confusing (Figure 10). However, there are many ways that something can be illogical without the error flag being raised by the visual system. As an example from ordinary language, consider the quote: “I write to you with my pistol raised and a sword in each hand.” This incensed 18th century politician has made a semantic error but, nevertheless, the sentence is grammatically correct. In our hunt for images with errors that give us pause, we therefore have to ask whether it is the visual system that pauses over the error or the cognitive system. Do we deduce that it is impossible, or *see* that it is impossible? This is not always obvious, and we have no way here to make an empirical test of the visual or cognitive nature of the error checking. But we will make a quick pass over several images (Figures 10 and 11) to see if there is anything interesting.

Figure 11 gives some examples where the flag being raised is not visual. On the left, a complete donut appears in the reflection on the toaster, but in front, it is half eaten. The mismatch does not concern vision, however, as the toaster continues to appear reflective even while its reflection is impossible. The confusion arises only at a cognitive level where we know that an object’s reflection should match the object. Vision apparently doesn’t care about this particular law of physics. Similarly, for the drawing by Oscar Reutersvard in the top middle, everything is locally fine, but the relations specified by each local occlusion are incompatible with the planar shape of the larger Z-shape piece. Once more vision doesn’t care. It doesn’t care either about the logical errors in Escher’s drawings. That is what makes them so entertaining—it takes some cognitive deduction to see where Escher’s scenes are impossible in 3D space. A similar example in spoken language would be Chomsky’s (1957) famous sentence “Colorless green ideas sleep furiously” (p.15). It is taken as grammatically correct albeit nonsensical, indicating that rules of grammar are independent of high-level meaning. Similarly, despite the violations of higher level “semantics” in the images of Figure 11, we still perceive them, confirming that there are rules for legitimate visual structure that do not depend on meaning either.

On the bottom right, the arrangement of the fixtures is certainly possible as it has actually been built, but some impossibility arises when we imagine them in use. In this case, though, it may very well be that vision simulates the concurrent usage and rejects it on the grounds of visual syntax—two bodies cannot occupy the same space at the same time. So, being puzzling is not, on its own, enough to be diagnostic of a visual grammar. An image can be puzzling without breaking visual rules, and it will require further digging and insights to come up with empirical tests that demonstrate when a broken rule is specifically visual. These demonstrations can then help discover what the rules of visual grammar may be.

## Acquisition of Grammar: Is Vision the “Ur-Language”

If there is a language of vision, there must be a way for the brain to learn that language and acquire its grammar. This seems particularly problematic for a language that lives within the brain—there are no other speakers to learn it from. It has to emerge on its own. Well, actually, not on its own. There is a steady stream of visual input to train a language and its grammar. Specifically, the regularities in the visual input would be sufficient to determine different classes of entities such as objects, actions, spatial, and temporal relations. Some things in the visual environment continue over time (objects), and others change the environment (actions), and so on. These regularities come from the physics of the world, but the visual system does not have to be specialized for physics; it only needs to be able to exploit regularities to construct the essentials of a language of vision. These regularities would also generate a grammar, specifying, among other rules, for example, that an action comes with an agent. Similar proposals of an emergence of structure from regularities have been proposed for other aspects of vision such as learning the metric structure of the world through regularities in photoreceptor responses as an object moves across them (Maloney & Ahumada, 1989) or how interactions with the world change the sensory input (Laflaquière et al., 2018; Philipona et al., 2003). In the proposal here, rather than just extracting the structure of space, language acquisition would have to extract the conceptual structure of the dynamic, visual world.

If a language of vision emerges from this Regularity Acquisition System, one fascinating question is whether this system then seeded first possibly gestural (Corballis, 1999; Donald, 1991) and then spoken language acquisition. That would make visual language the “ur” or original language. There is clearly an enormous gulf between how a visual language might be acquired from the physical environment and how a spoken language would be acquired from the speech environment. Nevertheless, the visual case could have created the foundation for the acquisition of grammar for spoken language. This would address the troublesome issue that there has not been enough time for the evolution of language (e.g., Corballis, 2017, 2019; Gregory, 1970; Sereno, 1991). In his 1970 radio broadcast, Gregory suggested that Chomsky’s (1965) deep structure had emerged much earlier to organize “the world in order to see it.” This deep structure was then able to support spoken language. Corballis (2017, 2019) similarly suggested that mental time travel and the common experience of the spatiotemporal world underlie our internal, universal grammar. He proposed that thought and then language had ancient origins in the original adaptation to the structure of space and time, conveniently allowing many million years for its evolution. Sereno (1991) also proposed that spoken language was built on existing functions that evolved for related purposes in vision: the serial assembly of glances in scene understanding and the ability to visually imagine past and future events.

This idea that vision is the “ur-language,” the origin of other languages that followed is perhaps too simplistic. What vision may have offered following languages is not a common deep structure or a template for a language but a template for acquiring a language. Visual grammar and spoken grammar probably have nothing in common, just like different spoken languages may have no overlap in their grammars. But both visual and spoken grammars may share the algorithms required to extract components and grammar from regularities in the visual and speech input streams, respectively.

If the acquisition of visual grammar did seed the mechanisms for the acquisition of grammar of spoken languages, we might find some evidence of the chain of development. To do so, we should look for similarities across systems, some fossils of visual structure that were retained in spoken structures, like remnants of ancient DNA. Jackendoff (1999) has made a similar suggestion about spoken languages, that modern languages carry archaic features from earlier protolanguages. To look for fossils of an even earlier visual language, we need to find some verbal grammatical form that seems arbitrary but exists as well in vision.

**Figure 12.**
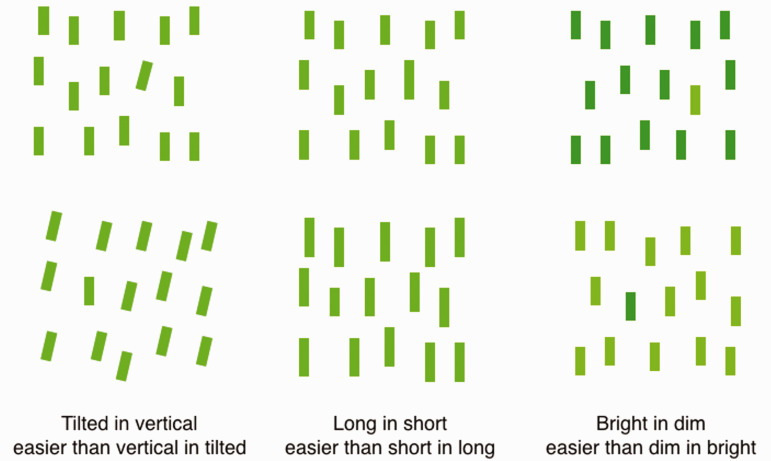
Asymmetry in visual search. It is easier to find certain targets among their opposites than the reverse: A tilted line can be found easily among vertical lines, a long line more easily among short lines than the reverse, and a bright line more easily among dim than the reverse (Treisman & Gormican, 1988). These asymmetries may be at the base of the similar asymmetries seen in spoken language (see text).*Note.* Please refer to the online version of the article to view the figure in colour.

Here is one example. In spoken language, adjectives describing physical properties often come in opposing pairs such as bright and dim, long and short, tilted and vertical, and wide and narrow. With these antonym pairs, one word is often the base term; it names the dimension. So, for bright versus dim, the base term is “bright,” and the dimension is brightness. For long versus short, the base is “long,” and the dimension is length. For tilted versus vertical, the base term is “tilt,” and the dimension is tilt. And so on. The terms in the pairs that are not the base term—dim, short—take longer to process in speech and are acquired later in development (e.g., Slobin, 1973). A similar asymmetry is seen in vision (Figure 12) for visual search where the equivalent of the base terms from spoken language is easier to find among their opposites than the reverse. This has been shown for tilted in vertical, long in short, bright in dim, and undoubtedly many others if they were to be tested. This result may be evidence that a particular conceptual structure from vision was retained in the structure of spoken language. Or it may be just a coincidence. A focused search for the fossils of a language of vision in the structures of spoken language may turn up additional evidence.

## Conclusions

Is there a language of vision? Clearly, even if there is, much of vision has nothing to do with the compressed messages the visual system would exchange with central processes. Nevertheless, this compressed messaging, the language of vision, is proposed here as the crucial link between the visual world and our conscious experience. I have based the components of visual language on the extremely obvious links from objects and actions, to nouns and verbs, and from spatial and temporal relations to prepositions. The ability to combine these components produces an unbounded set of descriptions and so gives the language of vision *compositionality*. Certainly, the neural patterns that represent objects and actions have no relation to the actual shapes or actions they describe. These neural symbols used by vision are therefore *arbitrary*. I also suggested that the verbs of vison can have tenses: past (object deformations, motion), present, and future (intentions, expected outcomes). One preposition, *behind*, gives another critical piece of evidence for a language: Vision can describe elements that are not visible (e.g., amodal completion). This is the property of *displacement,* the ability to describe things that are not present. There was also evidence that some high-level conceptual assignments are visual computations, not general cognitive inferences (causality, Rolfs et al., 2013; and causal past tense, Chen & Scholl, 2016). Finally, vision is full of *recursions*, another key component of a language—pictures within pictures, histories embedded in distorted objects. Overall then, the language of vision can be construed to have these four key properties for a language: compositionality, arbitrariness, displacement, and recursion.

A reviewer of this article has pointed out that many see vision as all about reception, the comprehension of input. In contrast, much of spoken language is about production, not just reception. However, the proposal of a language of vision changes the view of vision completely by specifically adding the production of the compressed, formatted messages that vision sends to the rest of the brain. Visual language has both production and reception sides where the reception includes not only incoming sensory information to be interpreted and described but also incoming messages and queries from the rest of the brain, arriving in the shared format of visual language. We could also imagine that action is another production of vision, but the link is not compelling. Actions are guided by vision but not produced by it the way the internal messaging of visual language is.

I proposed that attention is a principal actor in the language of vision. Attention not only selects salient items from the visual input but also constructs a description of visual events in a “language” format to send to the rest of the brain, and I suggested as well that the content of the message is the content of visual awareness. This view has three parts. The first takes attention as an active agent that does things. That is, of course, not new. Treisman (e.g., Treisman & Gormican, 1988) claimed that attention bound features together. Logan and Zbrodoff (1999) went further to claim that attention constructs propositional representations about the visual environment that are delivered to cognition, a proposal that is closely related to the one presented here. Some see attention as more restricted like a selection operation or a priority map (e.g., Itti & Koch, 2001) or a resource shared among competing processes (e.g., Kahneman, 1973; Norman & Bobrow, 1975) or a factor that improves sensitivity (e.g., Lu & Dosher, 1998; Yeshurun & Carrasco, 1998). Others think that attention is just a vague term covering many separate operations (Driver, 2001; Walsh, 2003). For the moment, I suggest that we should take attention to be the process that formats and sends out the description—at least, until a better segmentation of visual processes becomes available. Certainly, the elements that I suggested for the components of visual language—its nouns, verbs, and prepositions—mostly all require attention in order to be identified. The visual search evidence showed that targets that were familiar actions, the verbs of vision such as walking, or spatial relations such as above and behind (prepositions) were slow to find, indicating that slow, serial attentional processes were required. Only some objects, our nouns of vision, show the property of preattentive pop=out (shaded cubes, shaded bumps and dents, Enns & Rensink, 1990; Kleffner & Ramachandran, 1992). On the whole, most objects do not do this (e.g., Hochstein & Ahissar, 2002). So, at a very crude level, we can see that the basic elements of a language of vision live at a level where attention is required to select and label them.

The second part is the claim that the content of the message sent to the rest of the brain is the content of visual awareness. This meshes in a trivial way with the common view that attention is the gateway to awareness (e.g., Cohen et al., 2012) but also keeps the demands on the language of vision to a minimum. Awareness and attention are both low-capacity processes with a rate of throughput roughly similar to that of spoken language. The payoff of a language of vision that is simply the content of visual awareness is that we have access to it and can imagine tests of its structure. That is a payoff for the future though because, for the moment, it is not clear how we can dissect our stream of visual awareness to identify its structure, its grouping into events, and its specification of actions and objects and their features.

Finally, the idea of a language of vision requires a grammar. Nowak and colleagues (Nowak et al., 2002; Nowak & Komarova, 2001) have made the point that complicated descriptions require a formatted language with a grammar to overcome the memory and noise limitations of human brains. But I have made no proposals about what that grammar of vision would be like. We can imagine some elements of syntax in the structures of objects and the physics of occupying space and continuity in time. But making explicit proposals would be a large-scale project, well beyond the scope of this talk. Hopefully, we can collect more evidence of what breaks the rules of visual grammar before attempting to outline what it is. There are many books (e.g., Fodor, 1975, 2008; Schneider, 2011) and papers (Rescorla, 2009; Schneider, 2009) and even a Wiki site (https://en.wikipedia.org/wiki/Language_of_thought_hypothesis) on various versions of “mentalese,” the language of thought. There is no actual grammar for mentalese proposed in these sources either, so clearly this is a direction for future work. 

Before tackling that project, we should appreciate that even the vague notions about a language of vision presented here raise the intriguing idea that a visual language, if there is one, must have emerged well before spoken language and may therefore have been the seed for the development of spoken language. Not that spoken language would share the grammar of vision, any more than Chinese shares the grammar of Portuguese. Instead what they may share is the algorithms to extract grammar from the environment—the visual environment for visual grammar and the speech environment for spoken language. I offered one possible example of a fossil of visual grammar that may have been preserved in spoken language. We should look for more.

Many might think that all this discussion of rules and grammars is superseded by the powers of deep neural networks that have flourished since this talk was first presented (e.g., LeCun et al., 2015). This clash between networks and rules emerged earlier with the less capable connectionist models (Rumelhart & McClelland, 1987) where rule-based approaches to language were shown to have a higher level of explanatory power (Pinker & Price, 1988). This debate echoed the earlier one of Chomsky’s (1959) rules of syntax versus Skinner’s (1957) pattern learning. Now, however, more advanced networks can already learn to interpret spoken language (e.g., Sarikaya et al., 2014) and can understand visual events in order to guide autonomous vehicles (e.g., Li & Shi, 2019). Even so, a neural network that that creates competent descriptions of visual events is not a magic box whose mere existence explains human mental processes. It does, however, offer advantages in allowing access to its working parts. Deconstructing large-scale neural networks that have visual, language, and other modules might provide a proof of existence of a visual grammar used for communication between artificial modules. However, it may be a very different grammar from that of human vision and evidence instead for convergent evolution. Whatever the case, the visual system is itself a gigantic neural net, so the real prize will come from understanding how the human brain solves these problems.
